# A content analysis of research on technology use for teaching mathematics to students with disabilities: word networks and topic modeling

**DOI:** 10.1186/s40594-023-00414-x

**Published:** 2023-03-26

**Authors:** Mikyung Shin, Min Wook Ok, Sam Choo, Gahangir Hossain, Diane P. Bryant, Eunyoung Kang

**Affiliations:** 1 Department of Education, West Texas A&M University, Amarillo Center 332C, 720 S. Tyler, Amarillo, TX 79101 USA; 2 Daegu University, Gyeongsan, South Korea; 3 University of Minnesota, Minneapolis, USA; 4 University of North Texas, Denton, USA; 5 The Meadows Center for Preventing Educational Risk, The University of Texas at Austin, Austin, USA; 6 Joongbu University, Goyang, South Korea

**Keywords:** Research trends, Mathematics, Technology, Topic modeling, Students with disabilities, Word networks

## Abstract

The purpose of this study was to conduct a content analysis of research on technology use for teaching mathematics to students with disabilities. We applied word networks and structural topic modeling of 488 studies published from 1980 to 2021. Results showed that the words “computer” and “computer-assisted instruction” had the highest degree of centrality in the 1980s and 1990s, and “learning disability” was another central word in the 2000s and 2010s. The associated word probability for 15 topics also represented technology use within different instructional practices, tools, and students with either high- or low-incidence disabilities. A piecewise linear regression with knots in 1990, 2000, and 2010 demonstrated decreasing trends for the topics of *computer-assisted instruction*, *software*, *mathematics achievement*, *calculators*, and *testing*. Despite some fluctuations in the prevalence in the 1980s, the *support for visual materials*, *learning disabilities*, *robotics*, *self-monitoring tools*, and *word problem-solving instruction* topics showed increasing trends, particularly after 1990. Some research topics, including *apps* and *auditory support,* have gradually increased in topic proportions since 1980. Topics including *fraction instruction*, *visual-based technology*, and *instructional sequence* have shown increasing prevalence since 2010; this increase was statistically significant for the *instructional sequence* topic over the past decade.

## Introduction

Worldwide, the use of technology has rapidly innovated over the last several decades. With the advances in technological tools and systems in society, information has become easily accessible. It is clear that students are now immersed in technology from childhood and exposed to a vast number of technologies. Living in the digital age, in which students and teachers use a range of digital devices in the classroom, technology-mediated education methods have penetrated deeply into the field of education (Jones & Shao, 2011). Students are expected to select and use appropriate mathematical tools when engaging in mathematical activities in classrooms (National Governors Association Center for Best Practices and Council of Chief State School Officers, 2010). Mathematical tools can include any instructional materials and symbols that can be used to assist students in demonstrating their mathematical ideas and solving problems (Koestler et al., 2013). In particular, tools such as technology-incorporated methods can easily accommodate learning environments, optimizing meaningful access to classroom activities (Center for Applied Special Technology, 2018). The implementation of these technological tools in teaching and learning mathematics has been a recommended standard of the National Council of Teachers of Mathematics since 2000 (National Council of Teachers of Mathematics, 2000).

In addition to the proliferation of the use of educational technology in mathematics instruction, increasing attention has been paid to the use of educational technology to achieve diversity, equity, and inclusion in science, technology, engineering, and mathematics (STEM) education. According to the National Center for Education Statistics (2022), 7.2 million students, who comprise 15% of all students enrolled in public schools in the US, have a disability that adversely affects their academic performance and thus, need special education and related services. The use of technology to help students with disabilities has been an important area of research and practice in special education since the Technology-Related Assistance for Individuals with Disabilities Act was passed in 1988 and amended in 1994 (Blackhurst, 2005). One of the major goals of *America’s Strategy for STEM Education* (Committee on STEM Education, 2018) is to increase diversity, equity, and inclusion in STEM education for at-risk subgroups of students, including those with disabilities. Recently, there have been active movements (e.g., by the National Science Foundation and the Institute of Education Sciences) to advance the inclusion of students with disabilities in STEM education, thereby increasing their opportunities to use technology and eventually broadening their participation and equity in STEM education. Researchers have been disseminating their efforts to improve specifically the mathematical performance of students with disabilities using technology (Aspiranti et al., 2020; Bouck et al., 2020, 2022; García-Redondo et al., 2019; Kagohara et al., 2013; Kellems et al., 2020; Kiru et al., 2018; Nabors et al., 2020; Park et al., 2022; Satsangi et al., 2021a, 2021b; Shin et al., 2021b, 2023b; Xin et al., 2020). However, despite the increasing awareness of the need for diversity, equity, and inclusion in STEM education and the growing volume of research on supporting students who are struggling with mathematics, there is a lack of research on reviewing the topics of extant research in the field to identify the knowledge gap.

## Evolution of technology use for teaching mathematics

Starting in the late 1990s, educators implemented virtual manipulatives and technology-based three-dimensional interactive visual models for teaching mathematical concepts and skills (Moyer-Packenham & Bolyard, 2016). Virtual manipulatives were initially developed through Flash or Java Applet programs and were mainly available on computers in the late 1990s. With the introduction of HTML5 web standards, however, virtual manipulatives have become available on mobile devices and have been found to be effective in teaching mathematics to students with disabilities (Bouck et al., 2020, 2022; Satsangi et al., 2021b; Shin et al., 2021b, 2023b).

Since 2010, touch-based tablets and interactive apps have become ubiquitous instructional tools in classrooms, particularly when teaching mathematics to students with disabilities (Pitchford et al., 2018). Both native apps (one-time downloadable apps on information and communication technology [ICT] devices) and web apps (apps accessible with a Wi-Fi connection) have increased the number of learning opportunities and improved students’ mathematical achievements (Aspiranti et al., 2020; Kagohara et al., 2013; Ok & Kim, 2017). Thus, the development and implementation of other types of technology, such as adaptive intelligent tutoring systems (Xin et al., 2020), educational games (García-Redondo et al., 2019), video-based instruction (Park et al., 2019) Satsangi et al. 2021a); augmented reality (Kellems et al., 2020), and virtual reality (Nabors et al., 2020), have increased, becoming important tools in mathematics instruction.

## Previous content analysis on educational technology for students with disabilities

Over the past few decades, researchers have been implementing content analysis to deepen the understanding of research topics and trends in educational technology, including for students with disabilities. Specifically, Istenic Starcic and Bagon (2014) reviewed 118 studies on ICT-supported learning for the inclusion of people with special needs in seven educational technology journals (e.g., the *British Journal of Educational Technology* and *Computers and Education*) indexed in the Web of Science and published from 1970 to 2011. Applying content analysis, the authors found that more studies were published from 2006 to 2011 (44.7%) than during any other period; they identified the level of inclusion through an analysis of educational context (special schools, mainstream schools, and general support for life), addressing participant characteristics and research design. They further classified ICT interventions into technical interventions in the pedagogical or wider context (e.g., ICT access, teaching and learning methods, development, and testing of ICT solutions).

Adamu and Soykan (2019) also applied a content analysis in identifying trends in articles on the use of technology for people with dyslexia published in the Web of Science database from 2014 to 2019. A total of 46 studies were analyzed to determine the publication year and country, participant groups (e.g., students and adults), research methods (e.g., practical and experimental), teaching methods (e.g., e-learning and mobile-assisted learning), data collecting tools (e.g., quantitative and qualitative), and subject fields (e.g., informational technology and special education). The results revealed that the largest number of studies were published in 2018 and were conducted in the United States. The traditional teaching method had the highest frequency, followed by e-learning and mobile-assisted learning. Regarding the subject fields of the studies, medical and information technology had the highest frequency.

## Recent applications of text mining techniques

Recently, beyond reviewing the literature using the traditional approach of content analysis, researchers have applied text mining techniques to further consider nested data structures (tokens nested within words that were nested within a document) when reviewing unstructured text such as large bibliometric datasets. The text mining method enables researchers to examine how the words within each publication are associated, constructing the meaning for each topic. In an effort to display related text information, word networks have been used in the social sciences and been connected with bibliometric reviews (Li & Xiao, 2022; Marín-Marín et al., 2021). Here, networks are considered a collection of the elements and their connected joints, usually displayed as graphs (Newman, 2018). To identify word co-occurrences (pairs of words that occur together within a publication) and analyze topics that have emerged across the published literature over time, researchers have analyzed large bibliographical datasets and implemented machine learning–based text mining approaches (Sharma et al., 2019). Thus, a word co-occurrence network represents a list of words as nodes and edges connecting two co-occurring words (Garg & Kumar, 2018). The application of word co-occurrence using bibliometric data such as abstracts can help educators explore what two words appear in the same publication while comparing the degree of closeness between texts (Kim et al., 2018).

Topic modeling is another text-mining method to explore hidden patterns in unstructured data by automatically organizing a large volume of texts into a set of clusters (Papadimitriou et al., 2000). To analyze trends from large bibliographical datasets of published data, researchers have implemented topic modeling, which is unsupervised machine learning (Grimmer et al., 2022; Sharma et al., 2019). Topic modeling enables people to uncover semantic structures and apply statistical methods. Topic modeling has been widely applied to examine the evolution of topics in certain academic fields, such as education (e.g., Li & Xiao, 2022), science (e.g., Blei & Lafferty, 2007), and human–computer interaction (e.g., Jung & Yoon, 2020). The earliest and most frequently used topic modeling method is latent Dirichlet allocation (Blei et al., 2003), which is based on a three-level (i.e., word, topic, and document) hierarchical Bayesian model in which hidden topics are assigned to explain the observed words in a text corpus of documents. As an extension of the originally suggested latent Dirichlet allocation method and to explain complex topic relationships, researchers have suggested methods to examine changes in topics over time (Wang & McCallum, 2006) and the correlation between topics (Blei & Lafferty, 2007). More recently, the structural topic model (Roberts et al., 2014), which includes covariate information related to the characteristics of documents, was suggested as a more flexible and general model in the social science field. Structural topic modeling can uncover latent topics in texts by assuming each document as a mixture of correlated topics and incorporating document-level external covariates into the prior distribution of topics or words (Bagozzi & Berliner, 2018).

Recently, Chen et al. (2020b) reviewed 40 years of research on educational technology with approximately 4000 articles published in the journal of *Computers & Education*. Applying structural topic modeling, they detected statistically significant increasing trends in topics such as collaborative learning, e-learning, and social networks and communities. As a follow-up study, Chen et al. (2022) conducted another structural topic modeling on the use of artificial intelligence technologies in education, with more than 4500 publications published from 2000 to 2019. In general, there has been increasing research interest in using artificial intelligence in the educational field. The topics included intelligent tutoring systems for special education, natural language processing for language education, and the application of artificial intelligence engines (e.g., educational robots, affective computing, and recommender systems).

## Needs for the current study

Despite several efforts to examine the research trends on educational technology for students with disabilities (e.g., Adamu & Soykan, 2019; Istenic Starcic & Bagon, 2014), previous studies have several limitations when it comes to their methodology. First, the studies did not use various databases when searching the papers; they only included journals indexed in the Web of Science database. Istenic Starcic and Bagon (2014) even analyzed only seven educational technology journals indexed in the Web of Science database.

Second, the previous studies analyzed papers published in a short period of time (2014–2019; Adamu & Soykan, 2019) or published about 10 years ago (1970–2011; Istenic Starcic & Bagon, 2014). To include all possible papers, it is necessary to use various databases and expand the publication year period, including both dissertations and journal articles in the analysis.

Third, based on earlier studies by Chen et al. (2020b, 2022) regarding detecting topics and topic trends in educational technology, we extended this comprehensive review by examining the co-occurring words in each decade between 1980 and 2021. As described in the introduction section, there was especially a change and evolution in technology use in almost every decade during this time period. Thus, if we rely on a linear tread across the entire year, it is highly likely that we cannot capture a meaningful decreasing or increasing trend of topic proportions that could happen in a particular time period. Thus, we applied a piecewise model for discontinuity in slope and topic trends.

Finally, neither of the previous studies focused on technology for teaching mathematics to students with disabilities. Istenic Starcic and Bagon (2014) focused on ICT-supported learning for the inclusion of people with disabilities, and Adamu and Soykan (2019) analyzed the use of technology in dyslexia. Considering the importance of using technology to teach mathematics to students with disabilities, it is necessary to review and analyze studies focusing on this topic.

Furthermore, unlike Chen et al. (2020b, 2022), we manually screened all publications using the inclusion criteria (see below in the Methods), following the Preferred Reporting Items for Systematic Reviews and Meta-Analyses (PRISMA) guidelines (Page et al., 2021).

## Research purpose and questions

To fill the gap in the previous research, we have aimed to extend the scope of data collection and analysis of the text corpus while exploring co-occurring words and hidden research topics in the literature on teaching mathematics with technology to students with disabilities. In detecting word co-occurrences and latent topics that have emerged from sizable bibliometric datasets (e.g., abstracts) of several decades of research, semantic machine learning techniques can be beneficial in mitigating human subjective bias and reducing potential measurement errors (Chen et al., 2020a). This text mining approach allowed us to analyze the abstracts of publications from journal articles and dissertations found through an online database. Because the mean topic proportion across the corpus did not show trends in topics over time, we implemented a piecewise linear regression with three knots in 1990, 2000, and 2010 to examine how the research topics evolved in prevalence over time. The current study’s correlation patterns between publication year and topic proportion supported the hypothesis that topical prevalence depends on the document-level covariate, the publication year. The specific research questions (RQ) were as follows:

1. What word pairs are commonly observed in studies on teaching mathematics using technology for students with disabilities in each decade between 1980 and 2021?

2. What research topics emerged from the studies on teaching mathematics using technology for students with disabilities, and what words were highly associated with these topics?

3. How have these research topics evolved in prevalence over time?

## Methods

### Inclusion criteria

We used four inclusion criteria. First, the focus of the studies had to be on teaching mathematics using technology for students with disabilities in grades K-12. We included studies when the purpose of the study was to provide instructional experiences and perspectives for students with disabilities. Second, we included studies of any type (e.g., experimental, correlational, qualitative, survey), including systematic reviews and meta-analyses. Third, studies were journal articles or dissertations published in English between 1980 and 2021. Unpublished doctoral dissertations were included in the current review to cover the gray literature, reducing publication bias (Paez, 2017). When any dissertations were published as journal articles, we counted these as identical publications and included the latest published articles only to avoid any doubling of publication. Fourth, we included studies when they reported title and publication year with abstract. Considering that the abstract of a publication provides a comprehensive summary of the paper (American Psychological Association, 2020), we used abstracts as the text sources for text mining analysis. Thus, studies without abstracts were excluded. To perform text mining across published studies over the last 42 years, we implemented structural topic modeling and examined trends in the use of technology in mathematics instruction for students with disabilities. Thus, in the initial search, we chose the starting year of 1970. The year 1970 was when the US Congress enacted the Education of the Handicapped Act (P.L. 91–230), a federal law establishing a new Title VI (later known as Part B) for individuals with disabilities, as a way to encourage states to develop educational programs. However, there were only two studies in the 1970s that met the above inclusion criteria (Higgins, 1970; Koller & Mulhern, 1977). Considering the limited number of studies available in the 1970s, we decided to set the starting search year as 1980.

### Search strategies and extraction of bibliographic data

**Fig. 1:**
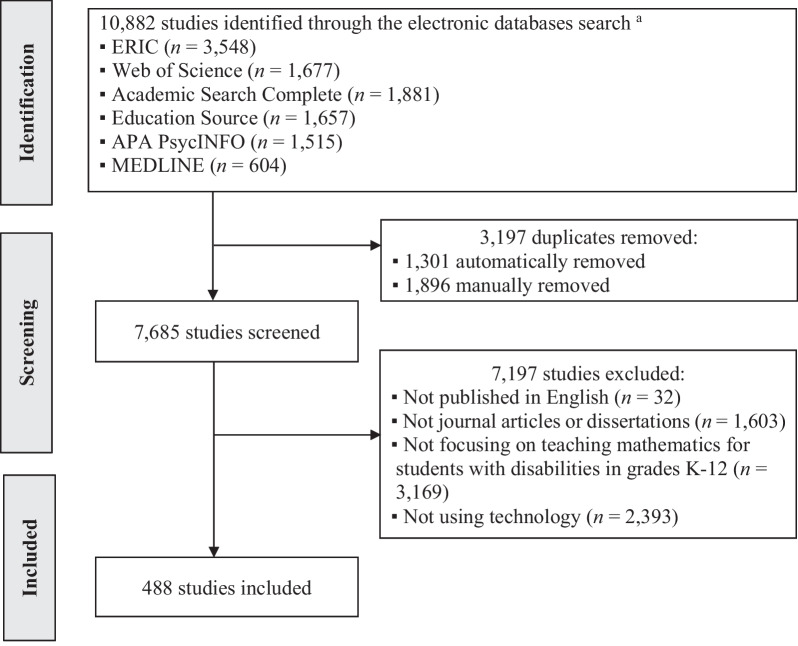
Flow diagram of identifying studies. ^a^ Search terms: (disab*) AND (math* instruct* OR math* intervent* OR math* teach*) AND (anchored* OR app* OR artificial* OR asynchronous* OR augment* OR blended* OR computer* OR digital* OR e-learning OR game* OR gamificat* OR iPad* OR mobile* OR smart* OR synchronous* OR tablet* OR technolog* OR three-dimensional* OR 3D OR universal design OR universal design for learning OR video* OR virtual* OR web*)

We adapted the PRISMA guidelines for article selection procedures (Page et al., 2021). As shown in Fig. 1, we first conducted an electronic database search of ERIC (*n* = 3548), Web of Science (*n* = 1677), Academic Search Complete (*n* = 1881), Education Source (*n* = 1657), APA PsycINFO (*n* = 1515), and MEDLINE (*n* = 604) for journal articles and dissertations published in English between 1980 and 2021, resulting in a total of 10,882 studies (see Fig. 1 for wildcard search terms used). When the first author exported the above search results as a bibliographic citation file (RIS) through the university library’s EBSCOhost Collection Manager, a total of 1301 duplicates were automatically removed from the online database by default. After exporting references to EndNote (EndNote Team, 2013), the team manually detected 1896 additional duplicates and removed these records from the lists.

We implemented the following procedure to extract bibliographic data of 7685 studies from EndNote (EndNote Team, 2013) to an Excel spreadsheet: (1) created a new output style that included bibliography records of reference type, author, year, title, keywords, and abstract; (2) removed carriage returns (unwanted paragraphs within each reference) in any field to display each reference in one row; and (3) copied formatted references into an Excel spreadsheet. Out of 7685 studies, we excluded 7197 studies for the following reasons: (a) not published in English (*n* = 32); (b) not journal articles or dissertations (*n* = 1603); (c) not focusing on teaching mathematics for students with disabilities in K-12 grades (*n* = 3169), or not using technology in mathematics instruction (*n* = 2393). As a result, 488 studies were included in the in-depth text mining.

To calculate inter-rater reliability on whether to include all the extracted studies, the first author reviewed 7685 studies and coded if each study met all four inclusion criteria. The other four co-authors independently double-checked all the metadata. We reached 98% agreement by taking the number of agreements and dividing by the total number of coding items multiplied by 100; after discussing the disagreed items, we reached 100% agreement on the included studies.

### Text preprocessing

To make the textual data appropriate for the algorithm, the research team preprocessed the textual data in three steps: (1) constructing a corpus by selecting a text column in the dataset (abstract) and combining texts with document-level variables (publication year); (2) constructing a token object by segmenting complex text into smaller words; and (3) constructing a document–feature matrix that displays the frequencies of features (i.e., tokenized words) for each document. As an initial step, the research team identified and manually extended 146 acronyms (e.g., “WPS” to “word problem-solving” and “VR” to “virtual-representational”). The team applied tokenization by changing texts to lowercase; converting accented characters to the American Standard Code for Information Interchange; splitting hyphens and tags; and removing punctuation marks, symbols, numbers, and separators.

Next, the team created customized dictionary objects that could be processed within the *quanteda* R package (Benoit et al., 2018). Before constructing dictionary word lists, we detected frequently co-occurring multiwords through the kwic() function, creating a list of multiword expressions (i.e., character vectors) with compound words and synonyms that depended on word order within the dataset. To avoid duplicating the exact words, we sequentially processed two different lists of word patterns with wildcard expressions (233 words list for the first dictionary and 181 words list for the second dictionary) through the dictionary() function.

Then, we removed commonly observed units (tokens) of words or patterns, that is, stop words, that were not distinct across documents. The team processed a predefined stop word lexicon using the English Snowball with a 175 stop words list (Porter, 2001), which is available through the *quanteda* package (Benoit et al., 2018); this led to 4627 distinct words across 488 publications. As a follow-up analysis, the team created a customized stop words list by calculating the *inverse document frequency* (*idf*) of each word. The *idf* of a term is a metric that shows the degree of distinction of words within documents, which is commonly defined using the following formula: *idf*_*i*_ = $\mathrm{log}\left(\frac{N}{{ {\text{df}}_{\text{i}} }}\right)$, where *df*_*i*_ is the number of documents in the corpus containing word *I,* and* N* is the total number of documents in the corpus (Hvitfeldt & Silge, 2021). We manually examined those below 5% in the *idf* ranking (i.e., 224 words) out of the 4627 unique words and went down the list until we identified distinct words. In this process, 107 of 224 words were eventually included, creating the remaining 117 as the customized stop words list. The lists of the extended acronyms, customized dictionaries, and stop word lists are shared on the online repository (Shin et al., 2023a).

### Word network analysis

In constructing and analyzing word networks, the research team identified co-occurring words within each publication regarding the use of technology in mathematics instruction for students with disabilities. To analyze data for each decade, we first filtered data by four different time periods (1980 to 1989, 1990 to 1999, 2000 to 2009, and 2010 to 2021). Then, applying the pairwise_counts() function in the *widyr* R package (Robinson & Silge, 2022), we counted the number of times each pair of words appeared together within a publication, occurring at least four times (1980s, 1990s, and 2000s) or 14 times (2010 to 2021). Thus, if two words (nodes) co-occurred in one publication, the nodes were connected with a line link (edge). We examined the importance of each individual word through a measure of degree centrality, assuming that influential and important nodes have higher neighbors (degrees) compared to other nodes with fewer degrees (Newman, 2018). To compare degree centrality (*C*) across networks with different numbers of edges and nodes, we normalized the values to be between zero and one, with one being the central node where all nodes are connected. For the normalized degree centrality calculation, we followed the formula embedded in the *igraph* (Csárdi & Nepusz, 2006) R package; *C* (*v*) = $\frac{{d}_{v}}{\left|N\right|-1}$, where *N* is the number of nodes in the network corpus, and *d*_*v*_ is the degree of node *v*. The visualization of the word co-occurrence network was completed through the *tidygraph* (Pedersen, 2023) and *ggraph* (Pedersen, 2022) R packages.

### Structural topic modeling

#### Basic structural topic model

To identify the topics from the 488 studies’ abstracts, we employed structural topic modeling (Roberts et al., 2014). Structural topic modeling allows “topical prevalence” (degree each document is associated with a topic) and “topical content” (associated words in each topic) to be correlated and affected by document-level covariates through a logistic-normal generalized linear model (Roberts et al., 2019, p. 2). However, for the current text mining, we included the document-level covariate (publication year) for the function of topical prevalence only. Document *d*, *d* $\in${1, …, 488}, is assumed to be a mixture of topics, *k* $\in${1, …, 15}. Each corresponding topic proportion *θ* denotes the probability that a topic is associated with each publication, and each observed word from a document (*w*_*d,n*_), where *n* ∈ {1, …, *N*_*d*_}, denotes that the *n*th word within a document has a corresponding topic assignment, randomly selected from a multinomial distribution given document-specific distribution over topics, *z*_*d,n*_ ~ Multinomial (*θ*_*d*_); a word is randomly selected from the corresponding multinomial distribution over terms conditional on the chosen topic, *w*_*d,n*_ ~ Multinomial (*β*_*d,k,v*_) (Grimmer et al., 2022; Roberts et al., 2019). A topic was considered a mixture of words selected among the 4510 distinctive terms in the current study. The sums of both topic proportions for a given document and word probabilities for a given topic were restricted to be one (Grimmer et al., 2022; Roberts et al., 2019). The formula for calculating the topic proportion and the document-specific distribution over words is available at https://mshin77.github.io/math-tech-sped.

#### Evaluating the optimal number of research topics

**Fig. 2:**
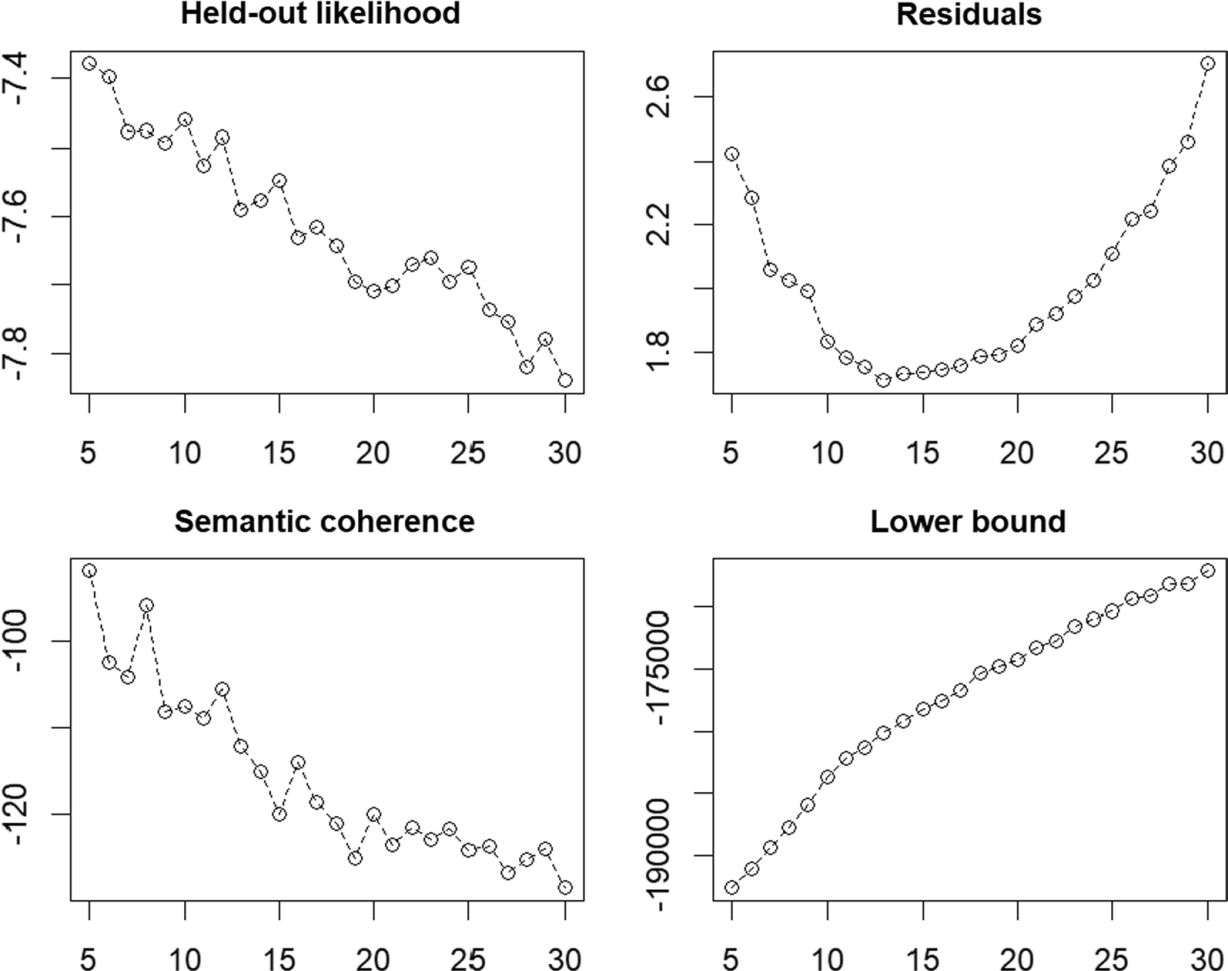
Model diagnostic by number of topics

Before conducting the topic modeling, the team first identified the optimal range of research topics, testing different numbers of topics against four different metrics. Applying a searchK() function in the *stm* R package (Roberts et al., 2019), we also evaluated multiple goodness-of-fit measures and identified the best fit for the data: (a) held-out likelihood: the log probability of topics assigned in the test set validating topics in the training set; (b) residuals: the difference between the predicted and expected topic predictions; (c) semantic coherence: co-occurrence of words in a topic; and (d) lower bound: the lower bound of the marginal log-likelihood (Rodriguez & Storer, 2020). As shown in Fig. 2, the diagnostic testing depicts the goodness of fit for each number of topics between 5 and 30. A topic number of 15 showed relatively low residuals, high semantic coherence, a maximized lower bound, and a high held-out likelihood.

#### Modeling topic prevalence

Before examining the evolution of topics, we calculated Pearson correlations between publication year and topic proportions of all topics to understand the overall trend—whether in a positive or negative direction. Then, considering different trend changes in topic prevalence over time, we estimated the topical prevalence parameter, applying a piecewise linear regression model for each of the 15 topics between 1980 and 2021. Specifically, we specified the intervals of trend changes bounded with three knots in 1990, 2000, and 2010 (Perperoglou et al., 2019).

To achieve continuity of fit in the data over the entire publication period, we restricted the model by adding a dummy coding for the conditions of the three knots and constructed the following piecewise linear regression formula: *topic proportion* = *β*_0_ + *β*_1_(*year* – 1980) + *β*_2_(*year* ≥ 1990)(*year* – 1990) + *β*_3_(*year* ≥ 2000)(*year* – 2000) + *β*_4_(*year* ≥ 2010)(*year* – 2010), where *β*_0_ = level (mean topic proportion) in 1980, centered at the year of 1980, *β*_1_ = trend (slope) in 1980s, *β*_2_ to *β*_4_ = trend change, and when *year* – *knot* = $$\left\{\begin{array}{l}0, \, {\text{if year}} < {\text{knot}} \\ {\text{year}}-{\text{knot}}, \, {\text{if year}} \ge {\text{knot}}.\end{array}\right.$$

In examining the relationship between the topical prevalence and publication year, the same model of topical prevalence (see above) was passed to an ordinary least squares regression using the estimateEffect() function in the *stm* R package (Roberts et al., 2019). Then, we extracted the *stm* effect estimate via the pointestimate() function in the *stminsights* R package (Schwemmer, 2021) and converted *stm* objects to a “tidy” format, which is a way of mapping data processed in the R environment through the *tidytext* R package (Silge & Robinson, 2016). Datasets, R codes, and detailed outputs were posted through an online data repository (Shin et al., 2023a).

#### Labeling topics and content validity

The research team manually labeled the topics that emerged from structural topic modeling. The first author labeled each topic by reviewing the most frequently observed words and representative publications. Then, four other co-authors in special education technology, mathematics, and computer science reviewed each labeled topic with associated words and publications and shared the recommended labels for each topic. Agreement for labeled topics was 93.33% (the team agreed on 14 out of 15 topics), and the research team reviewed each label and discussed it until a consensus had been reached.

## Results

### Descriptive summary of included studies

A total of 488 studies were published in English between 1980 and 2021 in teaching mathematics using technology for students with disabilities in K-12 grades; 416 studies (85%) were journal articles, and 71 (15%) were dissertations. The number of publications has increased over the last 42 years. The number of publications by period was 37 for the 1980s, 51 for the 1990s, 70 for the 2000s, 243 for the 2010s, and 87 for years of 2020 and 2021. Between 1980 and 2009, the minimum publication number per year was 1, and the maximum number was 12 per year (*median* = 5). However, since 2010, there has been a rapid growth in the publication number. In the period 2010–2021, the minimum publication number per year was 11, and the maximum number was 46 (*median* = 27).

### Co-occurring words over time (RQ1)

**Fig. 3:**
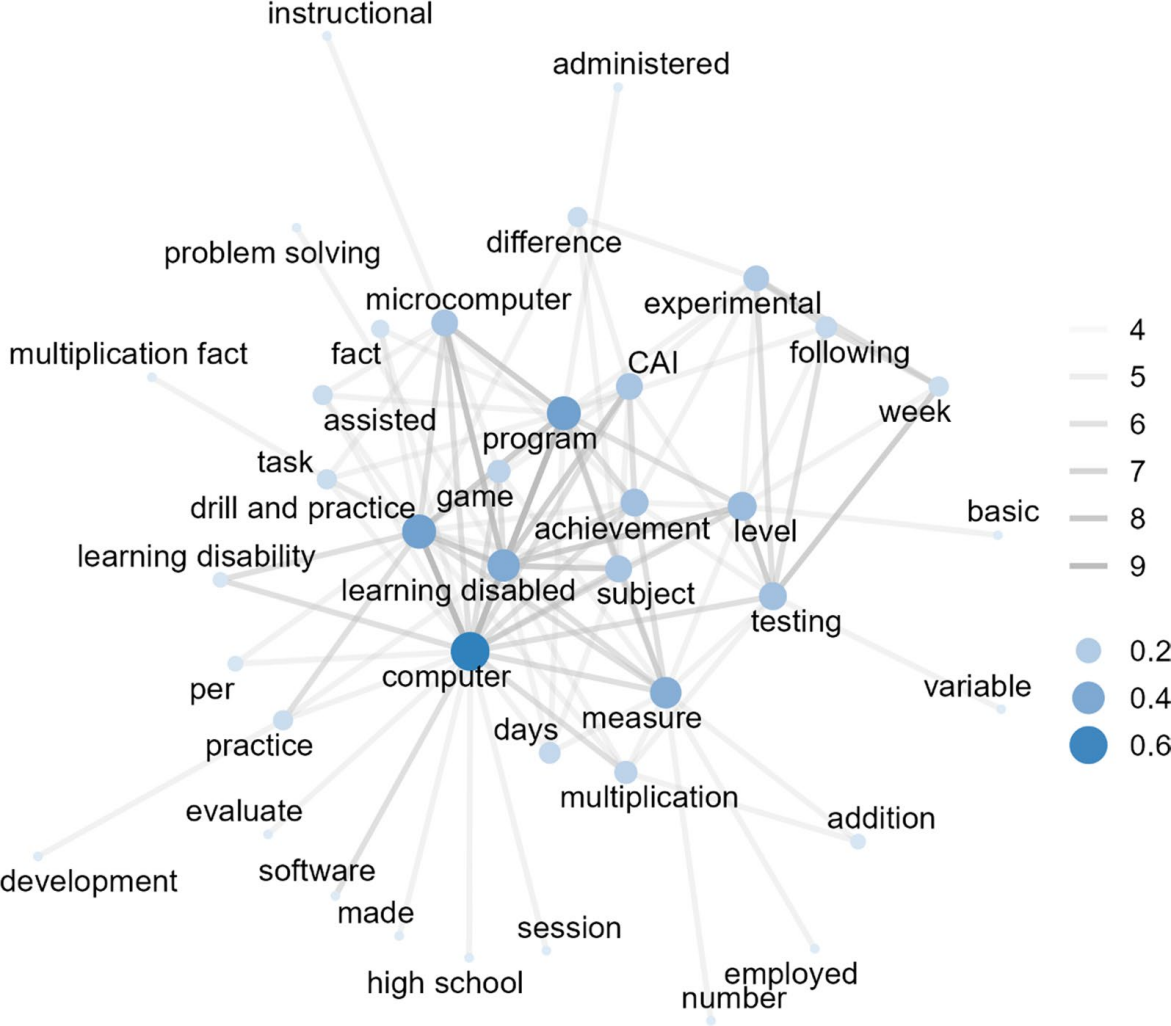
Co-occurring words (*n* ≥ 4) among studies published in 1980 to 1989 (39 nodes and 218 edges). CAI: computer-assisted instruction

In 1980 and 1989 (see Fig. 3), a total of 218 pairs co-occurred at least four times (39 nodes and 218 edges) out of 79,090 word pairs (*Min* = 1, *Max* = 9, *Median* = 1) in 37 studies. The word pairs with the highest co-occurrence (*n* = 9) were “learning disabled” with “program”, “computer” with “learning disabled”, and “computer” with “drill and practice”. A word with the highest level of degree centrality was “computer” (centrality = 0.63), followed by “program”, “drill and practice”, “learning disabled”, “measure”, “level”, “testing”, “achievement”, “microcomputer”, and “computer-assisted instruction (CAI)”.

**Fig. 4:**
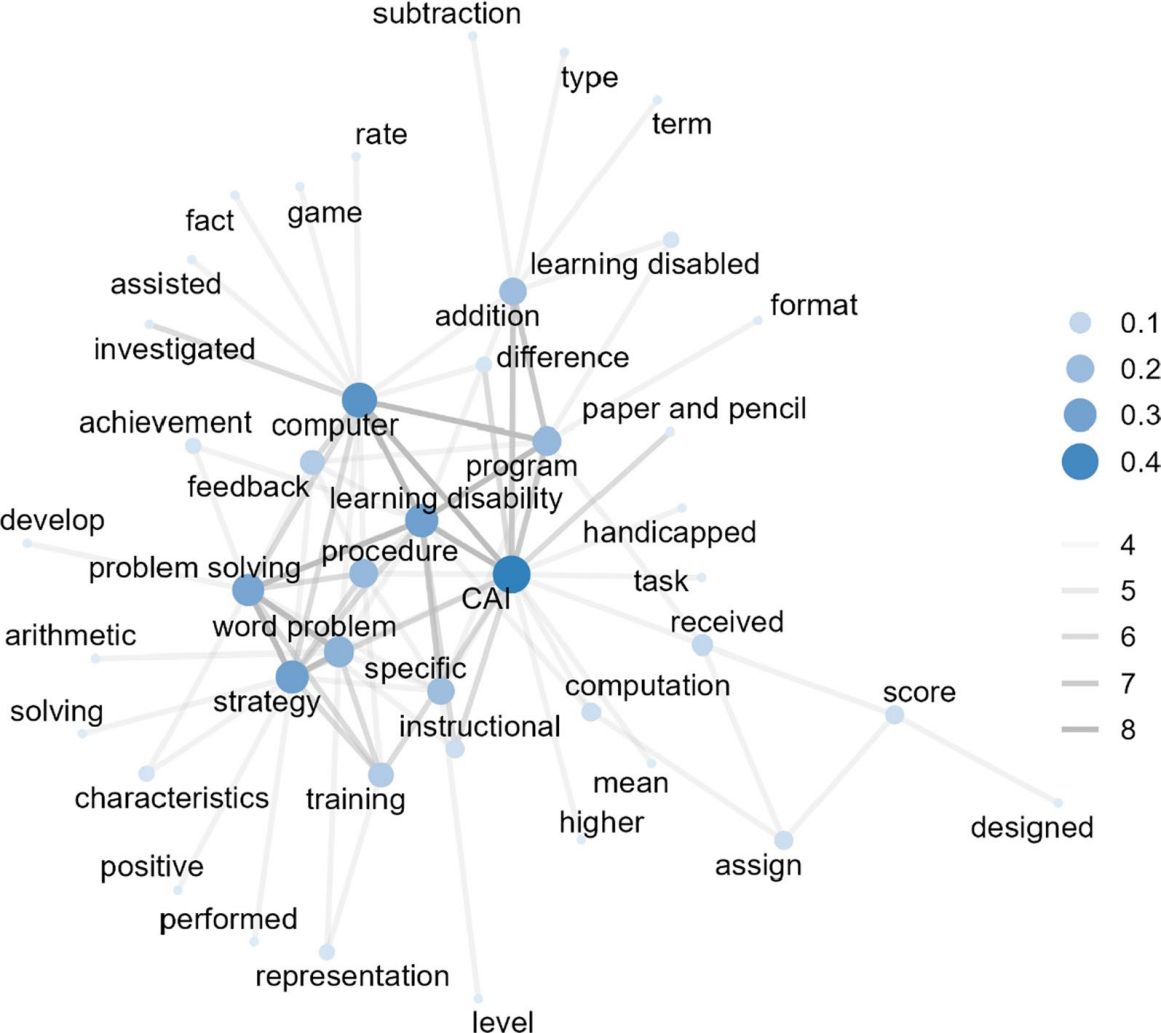
Co-occurring words (*n* ≥ 4) among studies published in 1990 to 1999 (43 nodes and 174 edges). CAI: computer-assisted instruction

In 1990 and 1999 (see Fig. 4), the co-occurrence frequency of 174 out of 71,736 word pairs (*Min* = 1, *Max* = 8, *Median* = 1) in 51 studies was greater than or equal to 4 (43 nodes and 174 edges). The word pairs with the highest co-occurrence (*n* = 8) included two sets: “strategy” with “problem solving” and “word problem” with “problem solving”. The next frequently co-occurring word pairs included “computer” with “program”, “learning disability” with “program”, “computer” with “CAI”, “learning disability” with “CAI”, “learning disability” with “computer”, “learning disability” with “problem solving”, and “word problem” with “strategy” (*n* = 7). The word with the highest degree of centrality was “CAI” (centrality = 0.43). The following words with relatively high degree centrality included “computer”, “strategy”, “learning disability”, “problem solving”, “word problem”, “program”, “procedure”, “addition”, and “specific”.

**Fig. 5:**
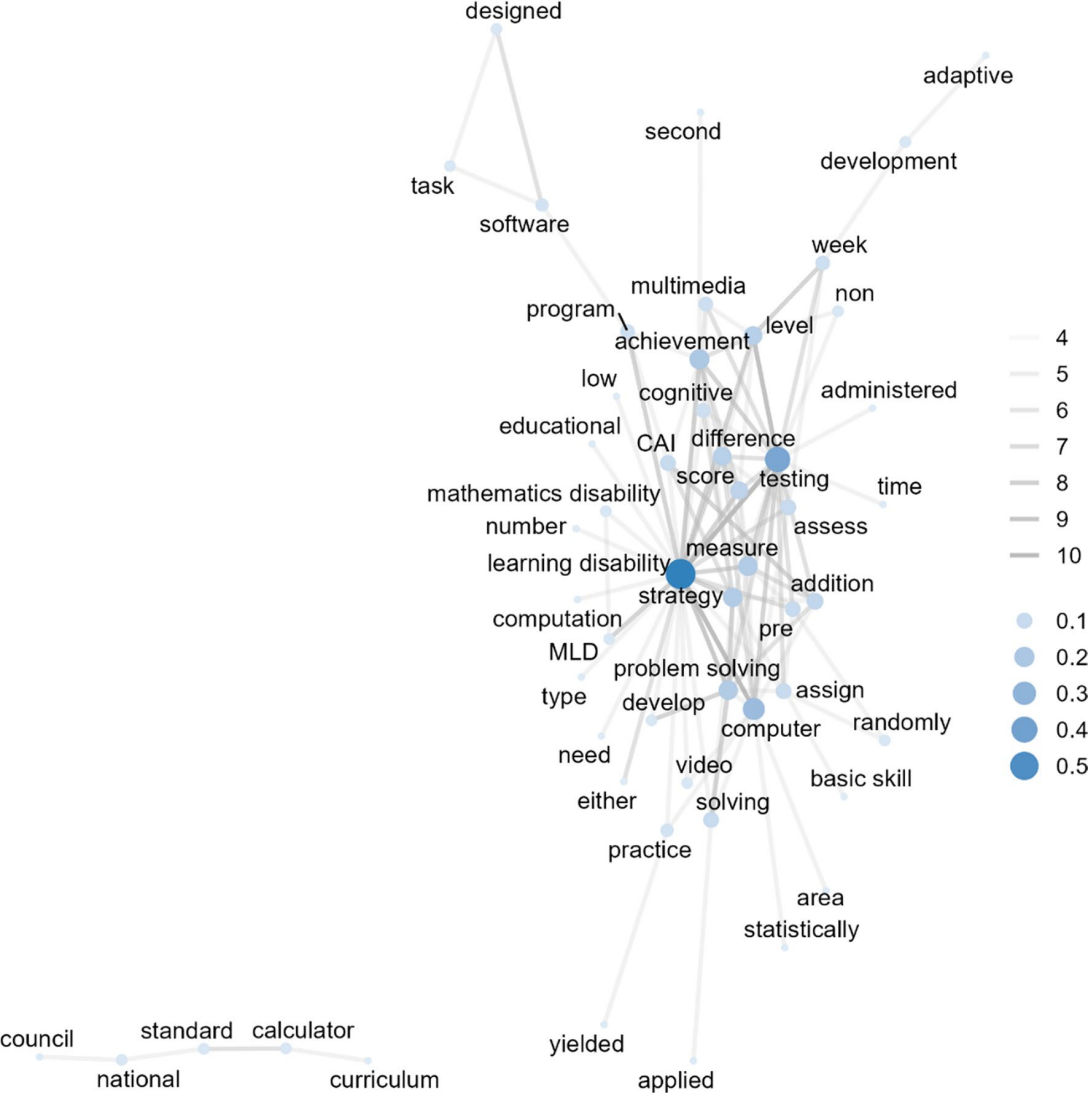
Co-occurring words (*n* ≥ 4) among studies published in 2000 to 2009 (52 nodes and 216 edges). CAI: computer-assisted instruction; MLD: mathematics learning disability

In 2000 and 2009 (see Fig. 5), the co-occurrence frequency of 216 out of 132,092 word pairs (*Min* = 1, *Max* = 10, *Median* = 1) in 70 studies was greater than or equal to 4 (52 nodes and 216 edges). The word pair with the highest co-occurrence included “learning disability” with “testing” (*n* = 10), followed by “learning disability” with “computer” (*n* = 9), “level” with “testing” (*n* = 8), and “learning disability” with “problem solving” (*n* = 7). The word with the highest degree of centrality was “learning disability” (centrality = 0.57), followed by “testing”, “computer”, “level”, “problem solving”, “addition”, “achievement”, “measure”, and “CAI”.

**Fig. 6:**
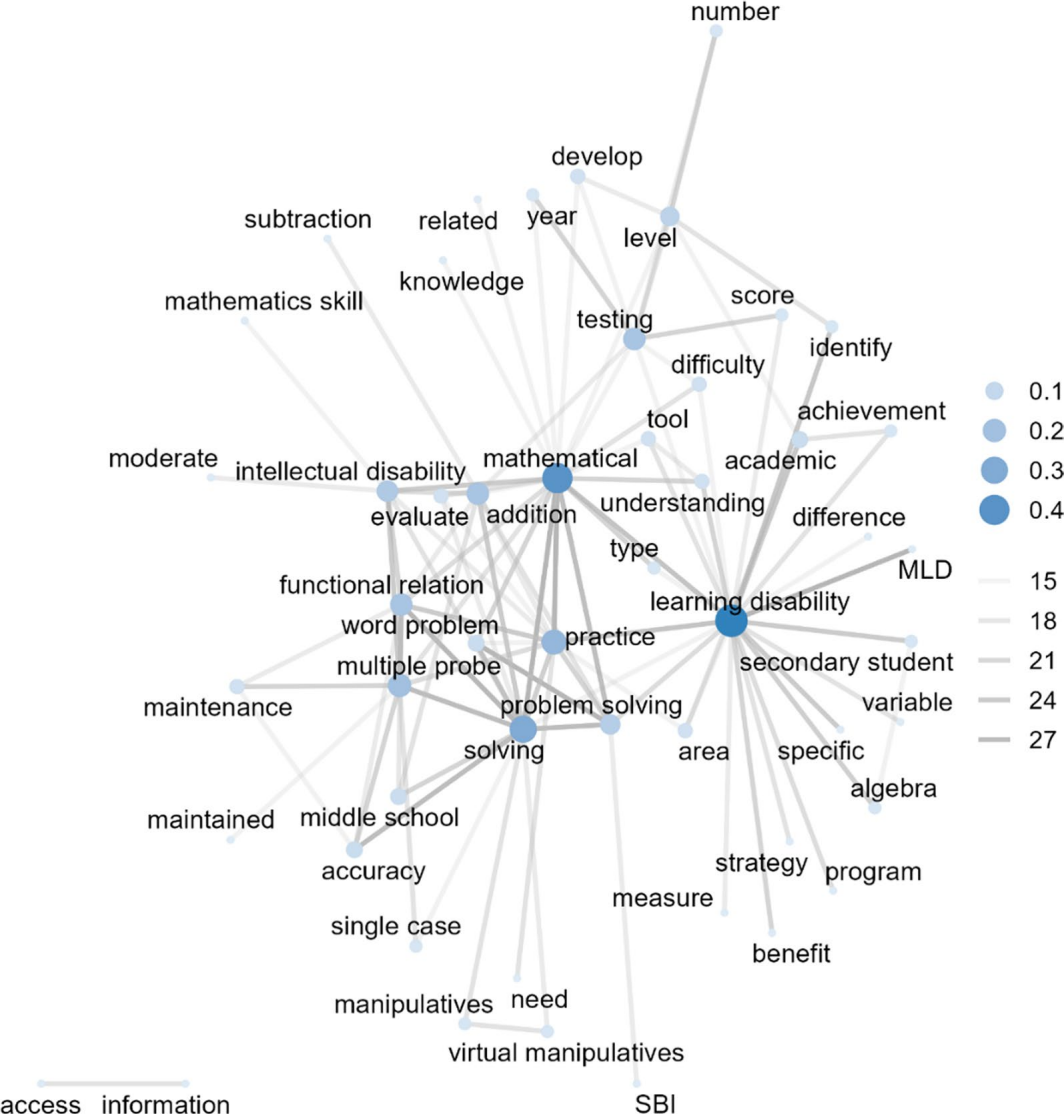
Co-occurring words (*n* ≥ 14) among studies published in 2010 to 2021 (51 nodes and 206 edges). MLD: mathematics learning disability; SBI: schema-based instruction

In 2010 and 2021 (see Fig. 6), the co-occurrence frequency of 206 out of 720,432 word pairs (*Min* = 1, *Max* = 27, *Median* = 1) in 330 studies was greater than or equal to 14 (51 nodes and 206 edges). The word pair with the highest co-occurrence was “functional relation” with “multiple probe” (*n* = 27), followed by “mathematical” with “practice”, “word problem” with “problem solving”, “MLD” with “learning disability”, and “mathematical” with “solving” (*n* = 26). The word with the highest degree of centrality was “learning disability” (centrality = 0.48), followed by “mathematical”, “solving”, “practice”, “multiple probe”, “functional relation”, “addition”, “testing”, “intellectual disability”, and “problem solving”.

### Emerged research topics and associated words (RQ2)

**Fig. 7:**
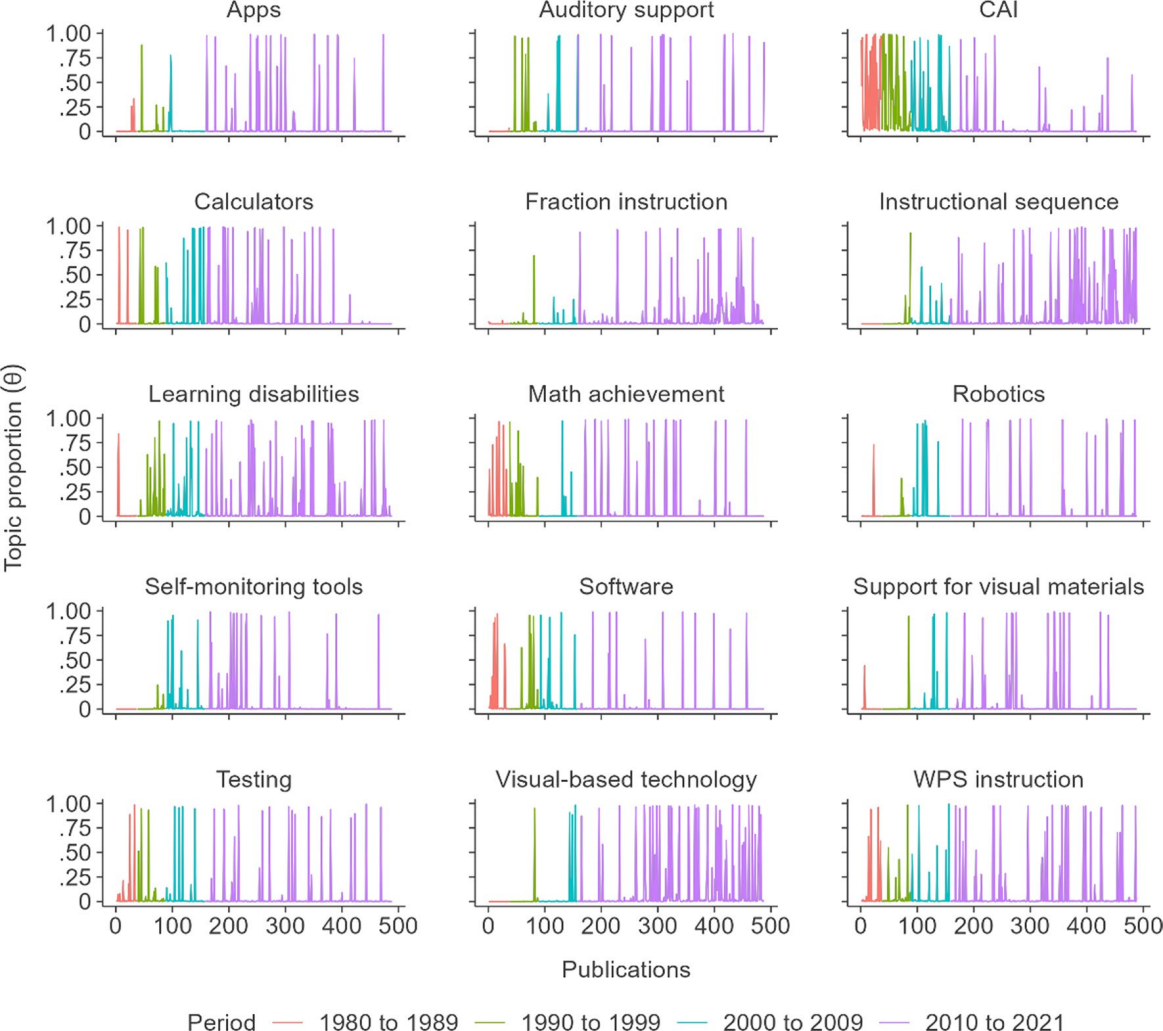
Distribution of topic proportions across publications. CAI: computer-assisted instruction; WPS: word problem-solving

Figure 7 shows the distribution of the probability that a given publication belongs to a given topic across publications. When the topic proportion is close to zero, this means that the particular publication has no association with that specific topic, and when the value is close to one, the target publication covers the topic to a great extent. Out of a total of 7320 topic proportions across the 15 research topics that emerged from the 488 publications, 6646 topic proportions (90.8%) were less than 0.1, and 304 topic proportions (4.2%) were greater than 0.9. Thus, this distinct probability distribution within a topic supports a clear classification for each topic.

Table 1 summarizes the associated terms (word probability per topic), labeled topics (topic proportion per document), and correlations between the topic proportions and the years of publication. The mean topic proportion ranged from .044 to .111 across the 15 labeled topics. Over the past 42 years, the five most frequently discussed topics were *CAI* (*θ* = .111), *instructional sequence* (mean *θ* = .096), *visual-based technology* (mean *θ* = .093), *learning disabilities* (mean *θ* = .093), and *word problem-solving instruction* (mean *θ* = .077). The other 10 topics investigated in the field included *calculators* (7.0%), *mathematics achievement* (6.3%), *testing* (5.7%), *robotics* (5.4%), *auditory support* (5.1%), *software* (5.0%), *support for visual materials* (4.8%), *apps* (4.7%), *self-monitoring tools* (4.5%), and *fraction instruction* (4.4%). These topics could be categorized further into instructional practices and assessment, educational technology tools, and disability types. To supplement the characteristics of each topic, we specified the representative study for each topic, that is, the study with the highest topic proportion for each research topic.

**Table 1:** Associated terms (word probability per topic), labeled topics (topic proportion per document), and correlations between topic proportion and year

Terms (*β*)	Topics (*θ*)	*r*	*p* value
CAI (.042), learning disabled (.018), program (.016), learning disability (.016), strategy (.014), addition (.013), computer (.013), procedure (.010), testing (.010), subject (.009)	CAI (.111)	− .49***	.000
maintenance (.017), framework (.015), practice (.015), intellectual disability (.011), VRA (.010), EBP (.010), mathematical (.010), instructional sequence (.009), mathematics skill (.009), explicit instruction (.009)	Instructional sequence (.096)	.25***	.000
virtual manipulatives (.035), manipulatives (.023), mathematical (.015), app (.013), solving (.013), learning disability (.012), algebra (.011), area (.011), tool (.010), concrete manipulatives (.010)	Visual-based technology (.093)	.22***	.000
learning disability (.062), problem solving (.021), game (.018), MLD (.018), strategy (.008), program (.008), specific (.007), identify (.006), educational (.005), digital (.005)	Learning disabilities (.093)	.04***	.000
problem solving (.032), computer (.027), mathematical (.025), word problem (.020), intellectual disability (.019), SBI (.012), solving (.010), video (.007), practice (.007), moderate (.007)	WPS instruction (.077)	.02*	.033
calculator (.050), accommodation (.021), testing (.019), assessment (.013), learning disability (.011), graphing calculator (.008), score (.008), curriculum (.007), difference (.007), access (.007)	Calculators (.070)	− .05***	.000
achievement (.032), level (.011), program (.010), measure (.009), learning disability (.007), score (.007), blended learning (.007), game (.006), engagement (.006), difference (.006)	Math achievement (.063)	− .09***	.000
testing (.015), application (.014), curriculum (.013), academic (.012), device (.011), iPad (.010), computerized (.010), behavior (.010), video prompting (.009), battery (.009)	Testing (.057)	− .03**	.006
measure (.021), robot (.021), device (.018), communication (.011), assistive technology (.010), speech generating (.010), level (.008), system (.007), task (.007), assessment (.006)	Robotics (.054)	.04**	.003
system (.017), blind (.011), shape (.010), relation (.008), sonification (.008), understanding (.008), tool (.007), environment (.007), geometry (.007), FXS (.006)	Auditory support (.051)	.02	.072
software (.034), geometry (.010), development (.010), number (.010), training (.010), computer (.008), game (.008), instructional (.007), level (.007), assessment (.007)	Software (.050)	− .14***	.000
information (.022), material (.012), image (.012), visually impaired (.012), knowledge (.011), STEM (.010), access (.008), graph (.008), graphics (.008), field (.008)	Support for visual materials (.048)	.07***	.000
app (.011), environment (.010), educational (.010), online (.010), pupil (.010), comparison (.010), need (.009), special (.009), time (.008), practice (.008)	Apps (.047)	.05***	.000
self-monitoring (.020), tool (.019), access (.017), assignment (.014), visual impairment (.014), digital text (.013), algebra (.011), homework (.011), phase (.011), textbook (.010)	Self-monitoring tools (.045)	.02*	.033
fractions (.086), accuracy (.026), solving (.025), middle school (.023), EAI (.021), functional relation (.019), addition (.018), instructional sequence (.017), virtual-abstract (.2017), session (.016)	Fraction instruction (.044)	.14***	.000

CAI: computer-assisted instruction; EAI: enhanced anchored instruction; EBP: evidence-based practice; FXS: fragile X syndrome; MLD: mathematics learning disability; SBI: schema-based instruction; STEM: science, technology, engineering, and mathematics; VRA: virtual-representational-abstract; WPS: word problem-solving

#### Instructional practices and assessment

Six topics (*CAI*, *instructional sequence*, *word problem-solving instruction*, *fraction instruction*, *mathematics achievement*, and *testing*) covered instructional practices and assessment. The *CAI* research topic has been the most frequently investigated over the past four decades. For example, as a representative study with the highest topic proportion, Nwaizu (1991) showed the effectiveness of teacher-assisted and computer-assisted instructional procedures among students with specific learning disabilities in mathematics. The words highly associated with *CAI* included “learning disabled”, “strategy”, “addition”, and “procedure”.

For the *instructional sequence* topic, Park (2019) implemented intervention packages with a sequenced combination of instructional methods, such as a virtual-representational-abstract instructional sequence with fading support or overlearning, to promote the maintenance skills of basic operations among students with disabilities. In the current topic modeling, words such as “maintenance”, “framework”, “virtual-representational-abstract”, and “evidence-based practice” were associated with the *instructional sequence* topic.

For the *word problem-solving instruction* topic, Schaefer Whitby (2009) showed the highest topic proportion; this study focused on the effects of a modified learning strategy (“Solve It!”) when solving multiple-step mathematical word problems among middle school students with high-functioning autism or Asperger’s syndrome. The highest associated words, such as “problem solving”, “schema-based instruction”, and “video”, supported the findings of previous syntheses and meta-analyses that schema-based instruction (Peltier et al., 2018), and video-based instruction (Kim & Xin, 2022) have been used for teaching word problems to students with disabilities.

In mathematics, *fraction instruction* has emerged as a distinctive research topic (Bouck et al., 2017). Other than “fractions”, the highly associated words, including “accuracy”, “solving”, “enhanced anchored instruction”, “instructional sequence”, and “virtual abstract”, show that studies have utilized various instructional practices when teaching fractions to students with disabilities.

Researchers have also focused on topics such as *mathematics achievement* in examining the relationships between education technology and mathematics achievement (McLeod, 2011) and *testing* in evaluating a computerized test battery for the diagnosis of learning disabilities (Billard et al., 2021). Of the highly associated words, “blended learning” for *mathematics achievement* and “computerized” for *testing* demonstrated the features of the measures and evaluations discussed within the included studies (Billard et al., 2021; Stewart, 2007).

#### Educational technology tools

Eight topics (*visual-based technology*, *calculators*, *software, apps*, *self-monitoring tools*, *robotics*, *auditory support*, and *support for visual materials*) covered the implementation of various educational technology tools for improving students’ mathematical performance. For the *visual-based technology* topic, representative studies with high topic proportions focused on technologies such as augmented reality, virtual reality, and virtual manipulatives. Miundy et al. (2019) examined the experiences of students with dyscalculia using augmented reality–based assistive digital technology. Altun and Kahveci (2019) evaluated the effects of virtual reality–based teaching material on geometry-related problem solving for students with learning disabilities. Prabavathy and Sivaranjani (2020) investigated the effects of virtual manipulatives on promoting basic arithmetic skills for students with developmental dyscalculia.

Regarding highly associated words, “accommodation”, “testing”, and “graphing calculator” demonstrated the purpose of *calculators* (Towers, 2018). For the consideration of *software*, words including “geometry”, “number”, and “computer” showed the targeted mathematics domain (Emprin & Petitfour, 2021). For the primary features of *apps*, “app” and “online” depicted the learning environment (Remata & Lomibao, 2021). Studies focusing on *self-monitoring tools* have addressed the usage of technology in expanding “access” to mathematics learning, such as using “digital text” (Bouck et al., 2013). These educational technology tools were found to be beneficial for teaching basic mathematics to primary school students with disabilities (Pitchford et al., 2018) and for improving the accuracy of mathematics homework (Falkenberg, 2010).

Furthermore, the research topic *robotics* highlighted the adapted and alternative function of a tool. Highly associated words, such as “robot”, “communication”, and “speech generating”, indicated the role of robotics in teaching mathematics to students with disabilities. A representative study by Adams and Cook (2014) also validated that robots have been used for the benefit of speech-generating functions in “hands-on” mathematics activities. Topics such as *auditory support* and *support for visual materials* were often investigated for students with visual impairments. The highly associated words for these topics were matched with representative studies: an interactive sonification of images in serious games as *auditory support* (Radecki et al., 2020) and textual materials (words, math expressions) describing visual images as *support for visual materials* (Emerson & Anderson, 2018).

#### Disability types

From the current topic modeling, only one topic (*learning disabilities*) was specifically related to disability type. Namely, the *learning disabilities* topic emerged as a separate research topic across the corpus. This topic covered words such as “problem solving”, “game”, “mathematics learning disability”, “strategy”, “problem”, and “digital”. These highly associated words can also be corroborated by the representative study conducted by Huscroft-D’Angelo et al. (2014), who investigated the impacts of a digital writing tool on improving students’ mathematical reasoning.

### Topic evolution over time (RQ3)

**Fig. 8:**
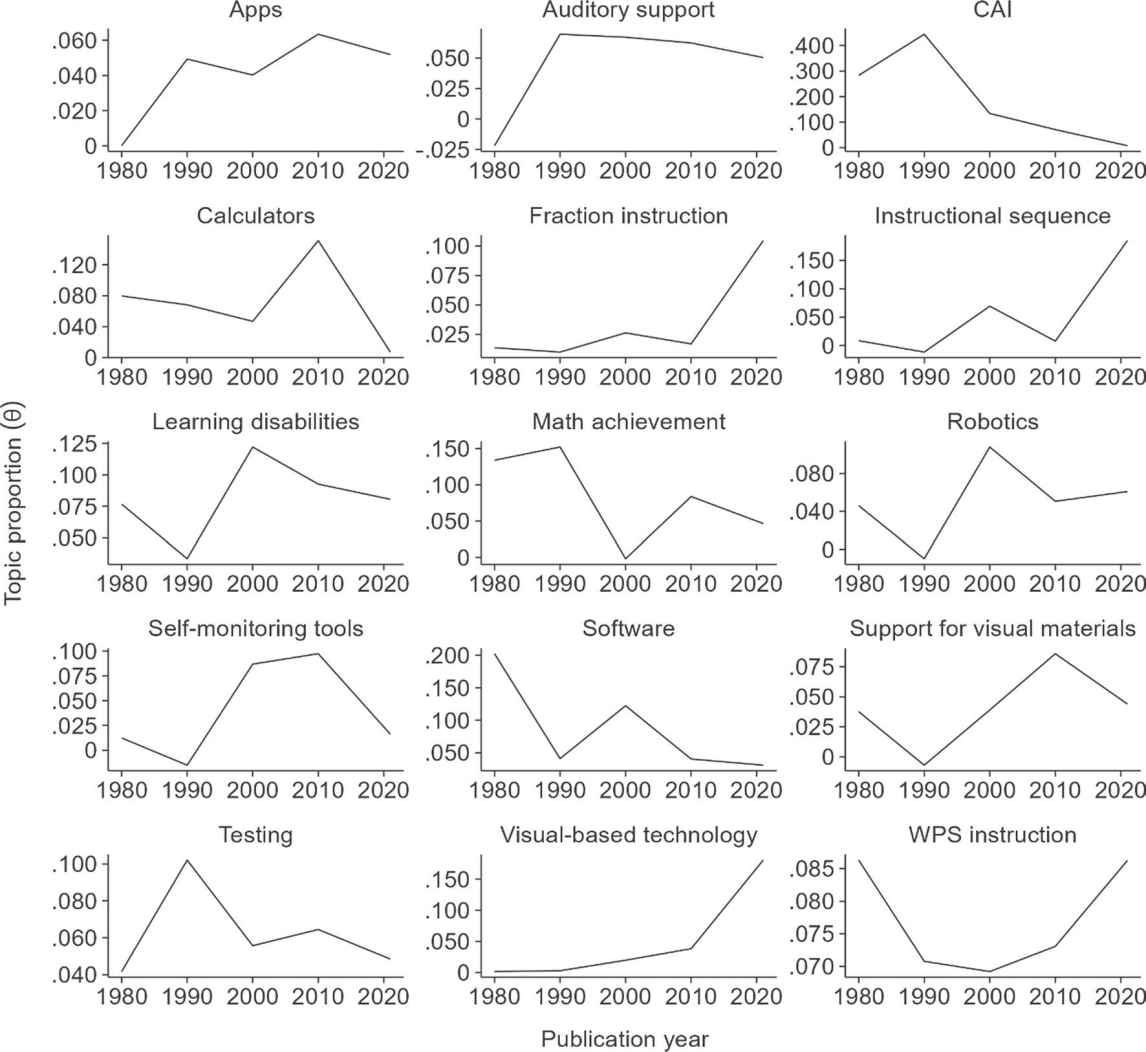
Piecewise linear trend of topic proportion for each topic. CAI: computer-assisted instruction; WPS: word problem-solving. The slopes of piecewise linear regressions are displayed above or below the line

To some extent, the degree of association between topic proportions and publications varied over time. Namely, the cases in which each publication was closely associated with a certain topic have shifted over time. This pattern of evolution of different topical prevalences over time demonstrates the effects of publication year as a document-level covariate. Figure 8 depicts the change in topic proportion per year during the four segmented periods of the 1980s, 1990s, 2000s, and 2010s, along with the more recent years of 2020 and 2021. Increasing or decreasing trends at the three knots (years 1990, 2000, and 2010) in the piecewise linear regression graph demonstrated the evolution of research interests and fluctuation in the publication numbers for a given topic. Table 2 summarizes the results of the piecewise linear regressions for each topic.

**Table 2:** Results of piecewise linear regressions

Variable	Estimate	SE	*t*	*p* value
Computer-assisted instruction				
Level in 1980	.270**	.095	2.830	.005
Trend in the 1980s	.017	.013	1.318	.188
Trend change after 1990	− .048*	.020	− 2.433	.015
Trend change after 2000	.025	.013	1.918	.056
Trend change after 2010	− .001	.008	− 0.111	.912
Instructional sequence				
Level in 1980	.005	.065	0.085	.933
Trend in the 1980s	− .001	.009	− 0.140	.889
Trend change after 1990	.009	.014	0.628	.530
Trend change after 2000	− .014	.011	− 1.320	.187
Trend change after 2010	.024**	.008	3.115	.002
Learning disabilities				
Level in 1980	.078	.082	0.950	.342
Trend in the 1980s	− .004	.011	− 0.377	.706
Trend change after 1990	.013	.018	0.736	.462
Trend change after 2000	− .012	.013	− 0.942	.347
Trend change after 2010	.004	.008	0.429	.668
Visual-based technology				
Level in 1980	.0001	.069	0.002	.999
Trend in the 1980s	.0003	.010	0.036	.971
Trend change after 1990	.001	.015	0.081	.936
Trend change after 2000	.001	.011	0.061	.951
Trend change after 2010	.010	.008	1.265	.206
Word problem-solving instruction				
Level in 1980	.084	.078	1.082	.280
Trend in the 1980s	− .002	.011	− 0.155	.877
Trend change after 1990	.002	.017	0.119	.905
Trend change after 2000	− .00003	.012	− 0.002	.998
Trend change after 2010	.001	.008	0.096	.924
Calculators				
Level in 1980	.085	.068	1.254	.210
Trend in the 1980s	− .002	.009	− 0.204	.839
Trend change after 1990	.0001	.015	0.009	.993
Trend change after 2000	.012	.011	1.058	.290
Trend change after 2010	− .023**	.008	− 3.108	.002
Mathematics achievement				
Level in 1980	.146	.079	1.846	.066
Trend in the 1980s	.001	.011	0.047	.963
Trend change after 1990	− .016	.017	− 0.935	.350
Trend change after 2000	.024*	.011	2.186	.029
Trend change after 2010	− .012	.007	− 1.744	.082
Testing				
Level in 1980	.037	.066	0.561	.575
Trend in the 1980s	.007	.009	0.722	.471
Trend change after 1990	− .011	.014	− 0.784	.433
Trend change after 2000	.005	.011	0.498	.619
Trend change after 2010	− .002	.007	− 0.258	.796
Robotics				
Level in 1980	.045	.061	0.743	.458
Trend in the 1980s	− .005	.008	− 0.657	.512
Trend change after 1990	.018	.013	1.375	.170
Trend change after 2000	− .018	.010	− 1.755	.080
Trend change after 2010	.007	.007	0.960	.338
Auditory support				
Level in 1980	− .023	.062	− 0.372	.710
Trend in the 1980s	.009	.009	1.027	.305
Trend change after 1990	− .010	.014	− 0.737	.462
Trend change after 2000	.001	.010	0.114	.909
Trend change after 2010	− .001	.007	− 0.187	.852
Software				
Level in 1980	.206**	.071	2.888	.004
Trend in the 1980s	− .017	.009	− 1.860	.063
Trend change after 1990	.027	.014	1.904	.057
Trend change after 2000	− .018	.010	− 1.777	.076
Trend change after 2010	.008	.006	1.260	.208
Support for visual materials				
Level in 1980	.039	.059	0.665	.506
Trend in the 1980s	− .005	.008	− 0.579	.563
Trend change after 1990	.009	.012	0.732	.465
Trend change after 2000	.001	.009	0.076	.940
Trend change after 2010	− .009	.006	− 1.406	.160
Apps				
Level in 1980	− .0003	.056	− 0.005	.996
Trend in the 1980s	.005	.008	0.607	.544
Trend change after 1990	− .006	.013	− 0.432	.666
Trend change after 2000	.003	.010	0.264	.792
Trend change after 2010	− .003	.006	− 0.403	.687
Self-monitoring tool				
Level in 1980	.011	.052	0.210	.834
Trend in the 1980s	− .002	.007	− 0.340	.734
Trend change after 1990	.012	.012	1.036	.301
Trend change after 2000	− .009	.010	− 0.873	.383
Trend change after 2010	− .008	.006	− 1.303	.193
Fraction instruction				
Level in 1980	.016	.051	0.314	.754
Trend in the 1980s	.0001	.007	0.017	.986
Trend change after 1990	.001	.011	0.087	.930
Trend change after 2000	− .002	.008	− 0.210	.834
Trend change after 2010	.007	.006	1.188	.235

SE: standard error. Bold font denotes statistically significant coefficients

^*^p < .05. **p < .01. ***p < .001

#### Generally decreasing trends

Although there were fluctuations in the slopes, there were decreasing trends for *CAI*, *software*, *mathematics achievement*, *calculators*, and *testing* topics. Table 1 shows the negative associations between topic proportions and publication year for these topics. In 1980, the topics of *CAI* and *software* showed average topic proportions (0.270 and 0.206, *p*s < .01, respectively) that were statistically significantly different from 0. Although not statistically significant, in 1980, the average topic proportion of the *mathematics achievement* topic (0.146, *p* = .07) was also relatively higher than the remaining topics. In the 1980s and 2000s, the trends of topic proportion for the *software* topic were reduced by .017 and .018, respectively, compared with those of each previous decade (*p*s > .05). Although there was a slight annual increase in the topic proportion (a .017 increase) in the 1980s, the slope for the *CAI* topic showed a decreasing trend since 1990; in particular, compared with the 1980s, the trend was decreased by .048 in the 1990s, which was statistically significant (*p* < .05). Furthermore, even though there was a statistically significant increase in the trend for the *mathematics achievement* topic in the 2000s compared with that of the 1990s by .024 (*p* < .05), after 2010, this again reduced by .012 (*p* = .08). There was also a slightly increasing topic proportion trend in the 2000s compared with the previous decade of .012 (*p* = .29) for the *calculator* topic; however, the topic proportions in other periods were largely decreasing trends. Regarding the *testing* topic, the topic proportion was estimated to increase annually by .007 in the 1980s (*p* = .47); yet in the following decades, the topic proportions followed generally decreasing trends.

#### Generally increasing trends

Although there were almost no existing publications in 1980, *apps*, *auditory support*, *fraction instruction*, *visual-based technology*, and *instructional sequence* have shown increasing trends since 1980. Table 1 depicts the positive associations between these topics and publication year. Specifically, regarding *apps* and *auditory support* topics, topic proportions increased by .005 and .009 (*p*s > 0.05), respectively, in the 1980s. Starting in 1990, there was no noticeable change for these topics; comparable topic proportions were maintained throughout the remaining decades for the *apps* and *auditory support* topics. Furthermore, the topic trends of *fraction instruction*, *visual-based technology*, and *instructional sequence* showed similar patterns. Despite a slight fluctuation before 2010 for the *instructional sequence* topic (trend increase by 0.009 in the 1990s and decrease by 0.014 in the 2000s compared with each previous decade), there was a statistically significant increase in the topic trend after 2010 by .024 (*p* < .01); thus, the average topic proportion was expected to increase by .018 during the years between 2010 and 2021. Both the topics of *fraction instruction* and *visual-based technology* showed limited topic proportions until the year 2010; there was .002 or less annual increase in the topic proportions. After 2010, there were relatively large trend changes: by .010 for *visual-based technology* and .007 for *fraction instruction*. Although these topic evolutions were not statistically significant, even after 2010, the increasing trends of these last topics demonstrated the latest technology being used in teaching mathematics to students with disabilities.

The topic proportions for the *support for visual materials*, *learning disabilities*, *robotics*, *self-monitoring tools*, and *word problem-solving instruction* topics showed increasing trends, particularly after 1990. Although statistically not significant, the average topic proportions of these topics increased after 1990. The topic proportion trends increased by .018 for the *robotics* and .013 for the *learning disabilities* topics in the 1990s, yet these were slightly reduced again in the 2000s (*p*s > .05). Regarding the topics of *support for visual materials* and *self-monitoring tools*, these topics also showed slightly increasing trends in the 1990s by .009 and .012, respectively, compared with those in the 1980s, and these increasing patterns persisted throughout the 2000s. Then, the trends for these topics declined after 2010. In the case of the *word problem-solving instruction* topic, after the initial decreasing trend in the 1980s, the topic trends increased slightly throughout the following decades; the trend change in the 1990s (.002) was relatively higher than in the other periods.

## Discussion

We conducted a content analysis via word networks and structural topic modeling exercise targeting studies on teaching mathematics using technology for students with disabilities. We examined 488 journal articles or dissertations published from 1980 to 2021. Our purpose was to identify co-occurring words and research topics investigated over the past 42 years. Using document–topic and topic–word distributions, we investigated the highly associated words for each topic and the topic evolution over time. By aggregating the results at the journal or dissertation level, we found that publications covered topics of different degrees both during a specific period and over time.

### Co-occurring words over time

Applying word networks, we sought to identify co-occurring words for journal articles and dissertations on teaching mathematics using technology for students with disabilities and to examine the empirical evidence for centrality among the co-occurring words. The central word of “computer” in the 1980s and its co-occurring words such as “CAI”, “microcomputer”, and “software” demonstrated the features of the computer in this earlier time in the education field. The word networks in this period validated the emergence and early efforts in the development of microcomputers and computer software focusing on number facts, such as “multiplication facts” and “addition”, for students with learning disabilities (Kelly et al., 1986; Palmer et al., 1985).

Unlike in the 1980s, “word problem” appeared to frequently co-occur with “CAI”, which had the highest degree centrality. This result highlighted the publication regarding the development of CAI that targeted teaching word problem-solving through strategic and procedural training in the earlier periods (Jaspers, 1991). Another noticeable word was “strategy”, which connected words such as “word problem”, “problem solving”, and “procedure”. These word networks showed the emphasis on instructional strategies and procedures in teaching word problems for students with disabilities in 1990s’ publications (Swanson, 1999).

In the 2000s and 2010s, the word “learning disability” showed the highest centrality. Although “learning disabled” and “handicapped” appeared in studies published in the 1990s, researchers started using person-first language around 2000 (e.g., students with learning disabilities instead of learning-disabled students), as well as applying videos, multimedia (Bottge et al., 2007), and calculators (Steele, 2007) in mathematics instruction and testing. In the 2010s, “solving” also showed a relatively high between centrality within the network, co-occurring with several other words, such as “virtual manipulatives”, “schema-based instruction (SBI)”, “single case”, and “middle school”. This indicates that, since the 2010s, publications on the use of virtual manipulatives have also included problem solving as their essential instructional component (Shin & Bryant, 2017). Furthermore, “intellectual disability” was also observed to co-occur with “problem solving”, which was connected to “SBI” and “middle school”. This indicates that SBI was frequently implemented when teaching mathematical problem solving to students with intellectual disabilities (Root et al., 2019). Additional words, such as “functional relation”, “multiple probe”, and “addition”, were connected to “middle school” and “intellectual disability” to indicate patterns of frequently observed research designs (i.e., single-case experimental designs) of mathematical domains (i.e., addition) for secondary school students (Bouck et al., 2021).

### Emerged research topics and associated words

Beginning with a relatively smaller number of publications in the 1980s, there has been exponential growth in the number of studies on teaching mathematics using technology for students with disabilities. In particular, the findings showed that researchers have largely focused on topics related to instructional practices and assessment. The high-probability words for each topic validated the instructional components and features used in each mathematics instruction. For example, associated words such as “learning disabled”, “strategy”, “addition”, and “procedure” applied for the *CAI* topic were aligned with the instructional design features of CAI, as analyzed by Ok et al. (2020), for teaching mathematical operations. Furthermore, other sets of high-probability words, such as “virtual-representational-abstract” and “evidence-based practice” for the *instructional sequence* topic, demonstrated the increased research focus on graduated instructional frameworks as evidence-based practices for teaching mathematics to students with disabilities (Jaspers et al., 2017). The highly associated words, such as “evidence-based practice” and “explicit instruction”, indicated that the instructional sequence was implemented based on the essential elements of explicit instruction (Bouck et al., 2020; Shin et al., 2021b).

Furthermore, the relatively high mean topic proportion for the *fraction instruction* and *word problem-solving instruction* topics also shows increasing interest in fractions and word problem-solving as primary concerns among students with disabilities. Because these two mathematics topics have consistently been a building block for successful mathematics in elementary and secondary mathematics (National Center for Education Statistics, 2019), it is assumed that researchers have been trying to investigate how to tackle these mathematical difficulties. Furthermore, since the release of the National Mathematics Advisory Panel’s report (2008) on highlighting the importance of teaching fractions as being a critical foundation of algebra and emphasizing students’ conceptual knowledge for understanding and solving mathematical word problems across topics, there have been an increasing number of intervention and review studies (Ennis & Losinki, 2019; Morano et al., 2020; Shin et al., 2021a).

Researchers have also focused on examining the roles and effects of various educational technology tools. As noticed among studies on *visual-based technology* topics, a growing number of researchers have applied virtual manipulatives, augmented reality, and virtual reality when teaching mathematics to students with learning disabilities (Altun & Kahveci, 2019; Prabavathy & Sivaranjani, 2020). In recent reviews and syntheses, researchers (Carreon et al., 2022; Nabors et al., 2020) have shown the positive outcomes and effectiveness of applying these technology tools for academic and behavioral aspects.

Although the overall research interest was low for the topics of *apps*, *self-monitoring tools*, *robotics*, *auditory support*, and *support for visual materials* compared with other research topics, these research topics should be noted. The associated word probabilities (e.g., “blind”, “visually impaired”, “speech generating”, and “digital text”) for these topics represented studies related to the support of students with low-incidence disabilities, such as visual impairment, blindness, and hearing impairment (Individuals with Disabilities Education Act, 2004; U.S. Department of Education, 2022). With a growing emphasis on promoting diversity, equity, and inclusion through technology in education, educational technology should consider a range of universal instructional supports for all learners and highly specialized assistive technology, such as text-to-speech, text/screen reader programs, and augmentative and alternative communication devices, for those who need specially designed functions (Kaczorowski et al., 2022).

### Topic evolution over time

In the current study, we found either generally decreasing or increasing topic trends. In particular, the topic trends addressed in the publications reflected the development and evolution of technology. For example, in the early 1980s, research topics such as *CAI*, *software*, *mathematics achievement*, *calculators*, and *testing* received attention, demonstrating relatively higher topic proportions or increasing trends, and these trends decreased over time. This research trend could be influenced by the emergence and rise of the personal computer and CD-ROM in the 1980s (Amankwah-Amoah, 2016). In the 1990s, the development of multimedia software was expanded through videodisc anchors (e.g., The Adventures of Jasper Woodbury) in mathematics instruction (Barron & Kantor, 1993). With the development of computers and software, these technologies were implemented in education research for students with disabilities at these times.

Although there were almost no available publications in 1980, research topics such as *apps*, *auditory support*, *fraction instruction*, *visual-based technology*, and *instructional sequence* have received attention since then. In particular, the increasing attention to *auditory support* and *apps* demonstrated the rise of ICT-supported learning for students with disabilities since 1990 (Istenic Starcic & Bagon, 2014). These findings have also highlighted the emergence of research applying technology-mediated instruction as enhanced anchored instruction (Bottge et al., 2018) and virtual manipulatives with a gradual systematic approach (e.g., virtual-representational-abstract instructional sequence; Bouck et al., 2017) or cognitive strategies within interactive computer application (Shin & Bryant, 2017) since 2010. Furthermore, although the words highly associated with the *visual-based technology* topic only included terms related to “virtual manipulatives” and “app”, the review of representative studies with the highest topic proportions also showed an increasing focus on augmented reality (Miundy et al., 2019) and virtual reality (Altun & Kahveci, 2019) over the past decade. Furthermore, driven by COVID-19 in 2020 and 2021, researchers have shown the increased application of online learning using these existing visual-based technologies in mathematics instruction (Bouck et al., 2022; Cox et al., 2021; Shin et al., 2023b; Tsuei, 2017).

Research topics such as *support for visual materials*, *learning disabilities*, *robotics*, *self-monitoring tools*, and *word problem-solving instruction* received increasing attention after 1990. These findings have indicated the increasing development of adaptive tools such as speech-generating robots (Adams & Cook, 2014) and audiovisual aids in digital texts (Bouck et al., 2013) during this time period. The *learning disabilities* topic was found to be the only disability type-related topic that emerged from the entire corpus. The increasing topic trend of *learning disabilities* over time shows a relatively higher degree of research focus on issues covering the identification and intervention of students with learning disabilities in teaching mathematics (Kiru et al., 2018; Lämsä et al., 2018; Ok et al., 2020) than others. Although other disability types did not appear as a distinctive research topic, words associated with the *word problem-solving instruction* topic also demonstrated that there was growing attention targeting students with intellectual disabilities, here incorporating video-based instruction (Saunders et al., 2018) and schema-based instruction (Root, 2016).

## Limitations and future research

The current study has several limitations. First, to examine the topics and trends in research on teaching mathematics through technology to students with disabilities, we explored journal articles and dissertations published in English between 1980 and 2021. Although we followed PRISMA guidelines (Page et al., 2021) and thoroughly screened publications from various online databases (e.g., ERIC, Web of Science, APA PsycINFO, and MEDLINE), we did not include some other types of publications, such as documents from social media (e.g., news and blogs) or gray literature (e.g., conference proceedings and unpublished works). These selection procedures could have limited the generalization of our findings by not capturing issues across domains beyond the academic field. Thus, future research should examine how the currently proposed topics and trends differ according to publication sources.

Second, although we applied several essential wildcard search terms (see Fig. 1) for the set of disabilities, mathematics, and technology, we did not include many other terms in the current database search. For example, the application of advanced technology in education was not fully considered through search terms, such as “flipped”, “online”, “mixed reality”, “machine learning”, and “deep neural network”. Furthermore, the aim of the current study was to review studies on the use of technology in teaching mathematics, but we excluded the emphasis on learning mathematics. Thus, in future research, a more extended search with comprehensive sets of the use of technology and mathematics learning should be included.

Third, in an effort to construct data-driven text preprocessing, the researchers developed user-defined dictionary objects and a customized stop words list that could be processed through the *quanteda* R package (Benoit et al., 2018). Although we took multiple and sequenced steps to develop customized dictionary objects (e.g., identifying frequently used multiword expressions) and the stop words list (e.g., removing commonly observed terms or patterns that are not distinct across documents), these lists could still be biased. Thus, in future research, the customized dictionary and stop words lists should be validated by external reviewers in multiple sectors, including linguistics, special education, technology, and mathematics.

Fourth, in the current study, we analyzed only the abstracts of the publications. Although an abstract is a comprehensive summary of a study (American Psychological Association, 2020), possibly it does not include all the key text contents in view of the publishers’ word limit guidelines. Additionally, dissertation abstracts usually include longer and more detailed summaries than journal article abstracts. These unequal word counts across documents could affect topic proportion and discovery. Thus, in future studies, researchers could extend the current study by analyzing keywords or even entire documents. Furthermore, word network analysis can be extended through the application of cluster-based topic modeling (ClusTop; Mu et al., 2022). Significantly, researchers can apply a word network graph with word embedding techniques or edge weights, depending on word embedding. This will enable researchers to systematically capture topical meaning in texts based on the network analysis’s community detection algorithms to automatically find the optimal number of subjects.

Finally, in the current structural topic model, only document-level covariates were considered when analyzing the associations between covariates (i.e., publication year) and topical prevalence. However, it is possible that topic-level covariates, such as types of studies that have been conducted (e.g., experimental and survey), types of instructional practices (e.g., fraction instructions and word problem-solving instructions), and educational technology tools (e.g., apps and calculators), which cover the categories of each topic label, could be of higher level affecting the topical prevalence across the corpus. Therefore, in future research, a structural topic model that considers covariates of topic and document levels should be investigated.

## Pedagogical and research implications

Three primary pedagogical and research implications were obtained from the current findings. The co-occurring word pairs that appeared within publications in each decade between 1980 and 2021 represented the usage and pattern changes of words in each time period. Although the use of computers through CAI was the most frequently researched topic in the 1980s and 1990s, especially for students with learning disabilities, we observed an exponential increase in research on mobile-friendly and online learning. Although disability types other than learning disabilities did not emerge as a distinctive research topic, the associated words probably supported the use of visual-based technology and videos for teaching word problems and basic mathematics to students with intellectual disabilities and autism spectrum disorder. These results indicate the need for research on students with other types of disabilities, particularly low-incidence disabilities. Future researchers can utilize educational technology applications, such as self-monitoring tools, robotics, auditory support, and support for visual materials, in their mathematics instruction for students with disabilities and validate the efficacy and roles of the interventions.

The results indicate that educators, researchers, and policymakers could use text-mining techniques to identify the essential features of instructional practices and tools. The associated words for each topic can be considered key components in designing technology-mediated mathematics curricula for students with disabilities. However, these data cannot suggest how effective each of the technologies is in improving the performance of students with disabilities in mathematics. Thus, in future studies, systematic reviews and meta-analyses are needed to synthesize the findings across studies, analyze deeper engagement with the issues unearthed in each study, and investigate how the inclusion of each component differs according to the student variables (e.g., grade and disability) and the field of study variables (e.g., technology). The public datasets and codes found in the current study will help future researchers replicate the suggested methodology, encouraging open and continuous communications in broadening the pathways for underrepresented groups of students in STEM and across disciplines.

Finally, the current study indicates research trends that align with the development of educational technology within the infrastructure of the industry. Specifically, accessible technology tools (e.g., speech-generating tools) are more frequently used in studies that target only students with visual or hearing impairments (Adams & Cook, 2014). However, in more recent years, researchers who teach students with other disabilities (i.e., learning disabilities, intellectual disabilities, and autism spectrum disorders) have applied virtual manipulatives, augmented reality, and virtual reality in their mathematics instructions (Bouck et al., 2022; Carreon et al., 2022; Nabors et al., 2020). In contrast to the findings of Chen et al. (2022), in the current study, the intelligent tutoring system did not emerge as a distinctive research topic. These findings indicate a lack of research on implementing other innovative approaches, including, but not limited to, the use of artificial intelligence or machine learning in mathematics instruction. In future research, partners across academia, industry, and other organizations need to collaboratively communicate to design, develop, and implement technology-mediated mathematics interventions that could extend learning access and promote the performance of students with disabilities in mathematics so that all learners can eventually succeed in their postsecondary education, careers, and independent living.

## Conclusion

Observing the discussed research topics and their evolution over the past 42 years can provide educators with a comprehensive understanding of the changes in research focus regarding the use of technology in teaching mathematics to students with disabilities. Because of the explosion of new sources and publications, a traditional content analysis through the manual coding of each document might appear to be consuming excessive time and effort. Text mining approaches, such as word networks and topic modeling, could prove effective in extracting meaningful categories and themes from large datasets, such as biblical datasets. By applying the suggested methods, researchers and policymakers can efficiently understand the key patterns addressed in published documents.

## Data Availability

The data and materials used and analyzed for the manuscript are publicly available at https://mshin77.github.io/math-tech-sped.

## Abbreviations

AR: Augmented reality
CAI: Computer-assisted instruction
EAI: Enhanced anchored instruction
EBP: Evidence-based practice
FXS: Fragile X syndrome
HTML: HyperText Markup Language
ICT: Information and communication technology
idf: Inverse document frequency
MLD: Mathematics learning disability
PRISMA: Preferred Reporting Items for Systematic Reviews and Meta-Analyses
SBI: Schema-based instruction
STEM: Science, technology, engineering, and mathematics
VR: Virtual reality
VRA: Virtual-representational-abstract
WPS: Word problem-solving
